# Confocal Microscopy in Skin Cancer

**DOI:** 10.1007/s13671-018-0218-9

**Published:** 2018-04-25

**Authors:** Verena Ahlgrimm-Siess, Martin Laimer, Harold S. Rabinovitz, Margaret Oliviero, Rainer Hofmann-Wellenhof, Ashfaq A. Marghoob, Alon Scope

**Affiliations:** 1 Abteilung für Dermatologie, Universitätsklinikum Salzburg, Paracelsus Private Medical University of Salzburg, Muellner Hauptstrasse 48, 5020 Salzburg, Austria; 2 Skin and Cancer Associates, 201 NW 82nd Ave #501, Plantation, FL 33324 USA; 3 Medical University of Graz, Graz, Austria; 4 Abteilung für Dermatologie, Universitätsklinikum Graz, Auenbruggerplatz 8, 8036 Graz, Austria; 5 Dermatology Service, Memorial Sloan Kettering Skin Cancer Center Hauppauge, 800 Veterans Memorial Highway, 2nd Floor, Hauppauge, New York, 11788 USA; 6 Medical Screening Unit, Sheba Medical Center, Ramat Gan 5262000, Sackler Faculty of Medicine, Tel Aviv University, Tel Aviv, Israel

**Keywords:** Reflectance confocal microscopy, Dermoscopy, Histopathology, Skin tumors, Facial macules, Non-melanoma skin cancer, Basal cell carcinoma, Nevi, Melanoma, Lentigo maligna

## Abstract

**Purpose of Review:**

Reflectance confocal microscopy (RCM) enables imaging of skin lesions at cellular level resolution at the bedside (in vivo) or in freshly excised tissue (ex vivo). This article provides an overview of strengths and limitations of non-invasive RCM in skin cancer diagnosis.

**Recent Findings:**

RCM features of common melanocytic and non-melanocytic skin neoplasms such as melanoma, actinic keratosis/squamous cell carcinoma, basal cell carcinoma, and nevi have been well defined and show good correlation with dermoscopic and histopathologic findings. Due to its technical properties, RCM is especially suitable for the examination of flat skin lesions.

**Summary:**

In vivo RCM has been shown to increase the accuracy of non-invasive diagnosis of common skin neoplasms and is a valuable adjunct to dermoscopy, particularly in cosmetically and functionally sensitive areas such as the face or the genital area.

## Introduction

The search to improve our clinical diagnostic accuracy for identifying skin cancer and to minimize unnecessary skin biopsies has led to the development of non-invasive imaging techniques. Among the non-invasive modalities, RCM imaging is unique in that it allows for the evaluation of the skin at the cellular-level. RCM evaluation can be performed at the bedside—in vivo, or on freshly excised tissue—ex vivo.

In vivo RCM enables the visualization of epidermis and superficial dermis in real time. While RCM has been used in a variety of skin conditions, its principal application remains the diagnosis of skin neoplasms, whereby the RCM optical sectioning of the skin simulates a “virtual biopsy.” This non-invasive approach is especially desirable for cosmetically sensitive areas such as the face or to help guide targeted biopsies within larger lesions. In addition, RCM enables repeated imaging of the same lesion over time, which can be used to determine the biologic nature of a lesion or for monitoring efficacy during and after non-invasive treatment. The ability to visualize dynamic processes by RCM (e.g., blood flow, inflammatory response) is useful for clinical as well as for basic dermatological research.

Ex vivo RCM imaging allows immediate evaluation of freshly biopsied tissue with almost no laboratory processing. Thus, ex vivo RCM may be used to expedite surgical margin assessment such as during Mohs micrographic surgery.

## Principles of Reflectance Confocal Microscopy

Confocal scanning microscopy was invented in 1957 by Marvin Minsky at Harvard University. However, an RCM device for skin cancer imaging was developed only in the 1990s and was driven by advances in optical and electronic technologies [1].

The basic principle of RCM is the use of a point source of light, which is tightly focused on a specific point in the tissue. The light is reflected back by certain tissue structures due to variations of refractive indices within the skin; specifically, melanin, hydrated collagen, and keratin are highly reflective skin components, which appear brighter on RCM images than the surrounding structures. Only light reflected back from the tissue focus point is allowed to enter the RCM detector through a pinhole-sized spatial filter, and to be processed by the dedicated software.

A commercially available RCM (Vivascope® 1500, Caliber I.D. Inc., Rochester, NY, USA) uses a near-infrared, low-power laser beam (830-nm diode laser, power up to 35 mW) for imaging. The laser beam is scanned in a two-dimensional grid over the skin to obtain a thin horizontal optical section, which is displayed as a grayscale image (“single RCM image”: 500 × 500 μm field-of-view). An automated stepper can be used to obtain up to 64 sequential images making a mosaic grid of 16 × 16 contiguous images in the horizontal plane (8 × 8 mm [2] field-of-view; “Vivablock”). By adjusting the focal length of the laser beam, a series of consecutive single RCM images can be stacked vertically (“Vivastack”), from the skin surface to superficial dermis, at the same point in the tissue. The dermoscopic picture of the lesion can be obtained with the RCM device and can serve as a gross map to guide RCM imaging of foci of interest within the lesion.

A handheld device (Vivascope® 3000, Caliber I.D. Inc., Rochester, NY, USA) enables imaging of curved anatomic sites, such as the nose or eyelids, and does not require fixation of the probe to the skin; the field-of-view is, however, limited to 1 × 1 mm [2] and the handheld device does not use a dermoscopic picture to guide RCM imaging.

The Vivascope® is equipped with a × 30 water immersion objective lens, providing a × 30 magnification and a lateral resolution of 0.5–1 μm for single RCM images. The axial resolution, which determines the section thickness, is approximately 3–5 μm, comparable to histopathological section thickness. Also analogous to routine histopathology, evaluation of RCM images is usually done first at “low magnification” (e.g., 8 × 8 mm mosaic is akin to × 2 magnification in histopathology) to examine overall lesion architecture and then at “higher magnification” by zooming in on the RCM mosaic to further examine architectural and cytomorphological details (at × 30 magnification).

The major limitation of RCM is the maximum imaging depth of approximately 250 μm, which usually correlates to the level of the papillary dermis or the upper reticular dermis. Another disadvantage is that assessment of nuclear details with RCM is significantly inferior to evaluation with hematoxylin and eosin histopathology.

## Reflectance Confocal Microscopy of Normal Skin

RCM imaging should follow a standardized protocol to enable accurate and reproducible image analysis. For this purpose, mosaic images (“Vivablocks”) of the suprabasal epidermis, the dermal-epidermal junction (DEJ), and the superficial dermis should be routinely acquired. Single images, “Vivastacks,” and RCM movies are taken in areas of special interest.

**Fig. 1:**
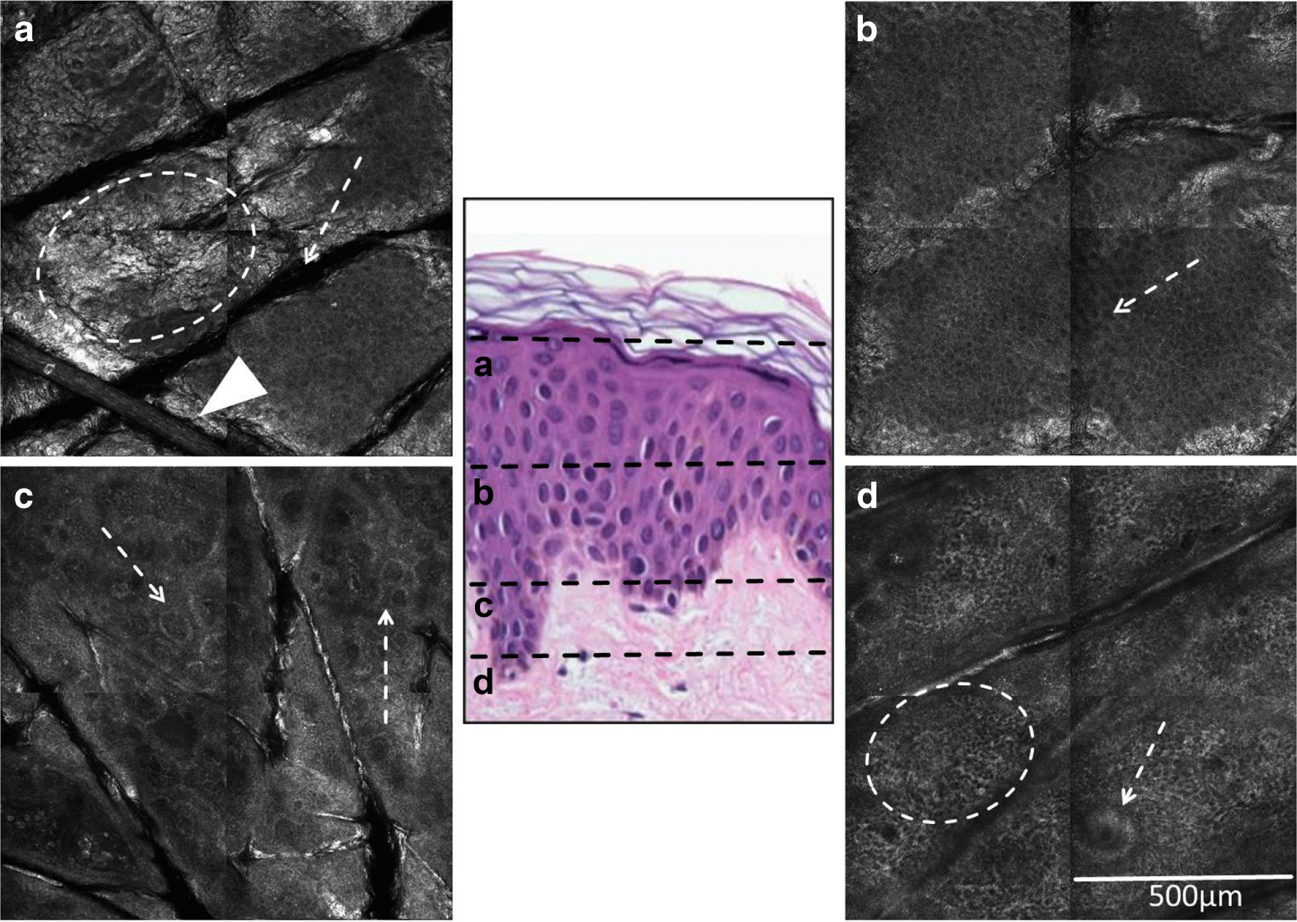
Horizontal RCM sections (1 × 1 mm) of normal skin at various imaging depths, as indicated on the corresponding vertically cut histological image. (A**)** Polygonal skin islands are separated by dark furrows (arrow); the furrows correlate to skin surface folds (dermatoglyphics). The stratum corneum presents as a “wrinkled”-appearing bright sheets (circle). A refractile long cylindrical tube correlating to a hair shaft is observed (arrowhead). (B) Regular honeycomb pattern of the granular and spinous layers seen with RCM. Dark holes of regular size, correlating to nuclei of keratinocytes (arrow) and bright lines with uniform thickness and reflectivity, correlating to cytoplasm of keratinocytes and intercellular connections, are seen. (C) Dark, round to oval dermal papillae surrounded by a slightly brighter rim of pigmented basal keratinocytes and melanocytes (“edged papillae”) are observed at the DEJ (arrows). (D) Within the papillary dermis, a web-like pattern of bright collagen fibers is seen (circle). The bright, centrally hollow structure (arrow) is a sweat duct; spiraling of the epidermal portion of the sweat duct (acrosyringium) through the epidermis can be seen when sequential images are obtained along the *z*-axis (“Vivastack”)

With RCM, the normal skin surface is seen as bright, large “wrinkled” appearing sheets of stratum corneum separated by dark furrows, which correlate to the skin folds (Fig. 1a). The overall RCM appearance of the spinous and granular layers has been called “honeycomb pattern” (Table 1), with bright lines of the honeycomb correlating to cytoplasm of keratinocytes and their intercellular connections and the dark holes correlating to the nuclei. In normal skin, the nuclei are regular in size and the lines show uniform thickness and reflectivity (Fig. 1b). Basal cells harbor a higher amount of melanin than other epidermal layers. In an undulating DEJ, basal keratinocytes will appear as disk-like bright cell aggregates when RCM sections at the suprapapillary plates (“cobblestone pattern”; Table 1), and as bright rings surrounding dark dermal papillae (“edged papillae”; Table 1) when RCM sections deeper, at the level of the dermal papillae (Fig. 1c). In a flattened DEJ, as seen in sun-damaged skin, basal keratinocytes may appear as aggregates or sheets of bright cells directly above the dermis; bright rings will not be seen. Within the dermal papillae, blood flow in capillary loops can be observed. Below the DEJ, a network of fibers and bundles can be seen within the papillary and superficial reticular dermis correlating to dermal collagen (Fig. 1d). The above described RCM features are the normal architecture and cell morphology of the skin that form the basis for recognizing tissue pathology on RCM. However, the appearance of normal skin will vary according to the patient’s age and skin color, the anatomic site being imaged and the degree of sun damage present [2].

**Table 1:** Key RCM features of normal skin by anatomic level

RCM feature	Definition
Superficial epidermal layers (granular-spinous layers)	
Honeycomb pattern	Normal pattern of the granular-spinous layers formed by bright polygonal outlines of keratinocytes (cytoplasm and intercellular connections) with dark central nuclei.
Cobblestone pattern	Normal pattern of basal keratinocytes at the suprapapillary plates and a variant of the normal pattern of the granular-spinous layers in darkly pigmented skin; bright round cells without a visible nucleus (pigmented keratinocytes) are closely set, separated by a less refractive polygonal outline.
Dermo-epidermal junction	
Edged papillae	Dermal papillae demarcated by a rim of bright cells (pigmented basal keratinocytes and melanocytes).
Superficial dermis	
Collagen	Bright fibrillar structures that appear finely reticulated, forming a web-like pattern, or as thicker bundles.

## RCM of Non-melanoma Skin Cancers

Non-melanoma skin cancer (NMSC) tends to occur on a background of significantly photo-damaged skin. Patients can have numerous basal cell carcinomas (BCCs) or squamous cell carcinomas (SCCs) concomitantly, and those need to be distinguished from the background solar lentigines, lichen planus-like keratoses, and seborrheic keratoses. Dermoscopy has limited utility when it comes to the diagnosis of pink, erythematous, non-pigmented skin lesions. To this end, RCM has been emerging as a promising imaging tool that can help diagnose, with high degree of specificity and sensitivity, these solitary pink lesions [3, 4].

### Reflectance Confocal Microscopy of Actinic Keratosis and Squamous Cell Carcinoma

With RCM, actinic keratosis (AK) shows an irregular and thickened stratum corneum, which correlates to the ortho- and parakeratosis seen on histopathology [5]. Focally, dark nuclei are visualized on RCM within the stratum corneum, correlating with parakeratosis on histopathology. The stratum granulosum and spinosum display on RCM pleomorphism of keratinocytes—the cells are irregularly crowded, vary in size and shape; these findings create an irregular honeycomb pattern with lines that vary in width and level of brightness, and with dark nuclei that vary in size or that are obscured (Table 2). Within the papillary dermis, abnormal collagen bundles are seen that are wider than normal collagen bundles and that appear clumped, correlating with solar elastosis on histopathology. Small bright stellate dots, correlating to inflammatory cells, may also be detected within the dermis.

**Table 2:** Key RCM features of non-melanoma skin cancers

RCM feature	Definition
Actinic keratosis and squamous cell carcinoma	
Irregular honeycomb pattern	Abnormal pattern of the granular-spinous layers formed by bright cellular outlines, which vary in size and shapes and in the thickness and brightness of the lines.
Disarranged pattern	Focal or diffuse loss of the normal patterns of the granular-spinous layers (honeycomb or cobblestone).
Round blood vessels	Dilated blood vessels within the dermal papillae that run perpendicular to the horizontal confocal plane of imaging.
Basal cell carcinoma	
Streaming	Basal or spinous keratinocytes that appear to be focally elongated and distorted into alignment along the same axis.
Tumor islands	Round to oval, cord-like or lobulated structures at the level of DEJ or superficial dermis that can be either bright structures, well demarcated by a surrounding dark cleft, or areas darker than the surrounding dermal collagen (“dark silhouettes”).
Linear blood vessels	Branching and tortuous dilated blood vessels in the superficial dermis that run parallel to the horizontal (*en face*) RCM plane of imaging.

**Fig. 2:**
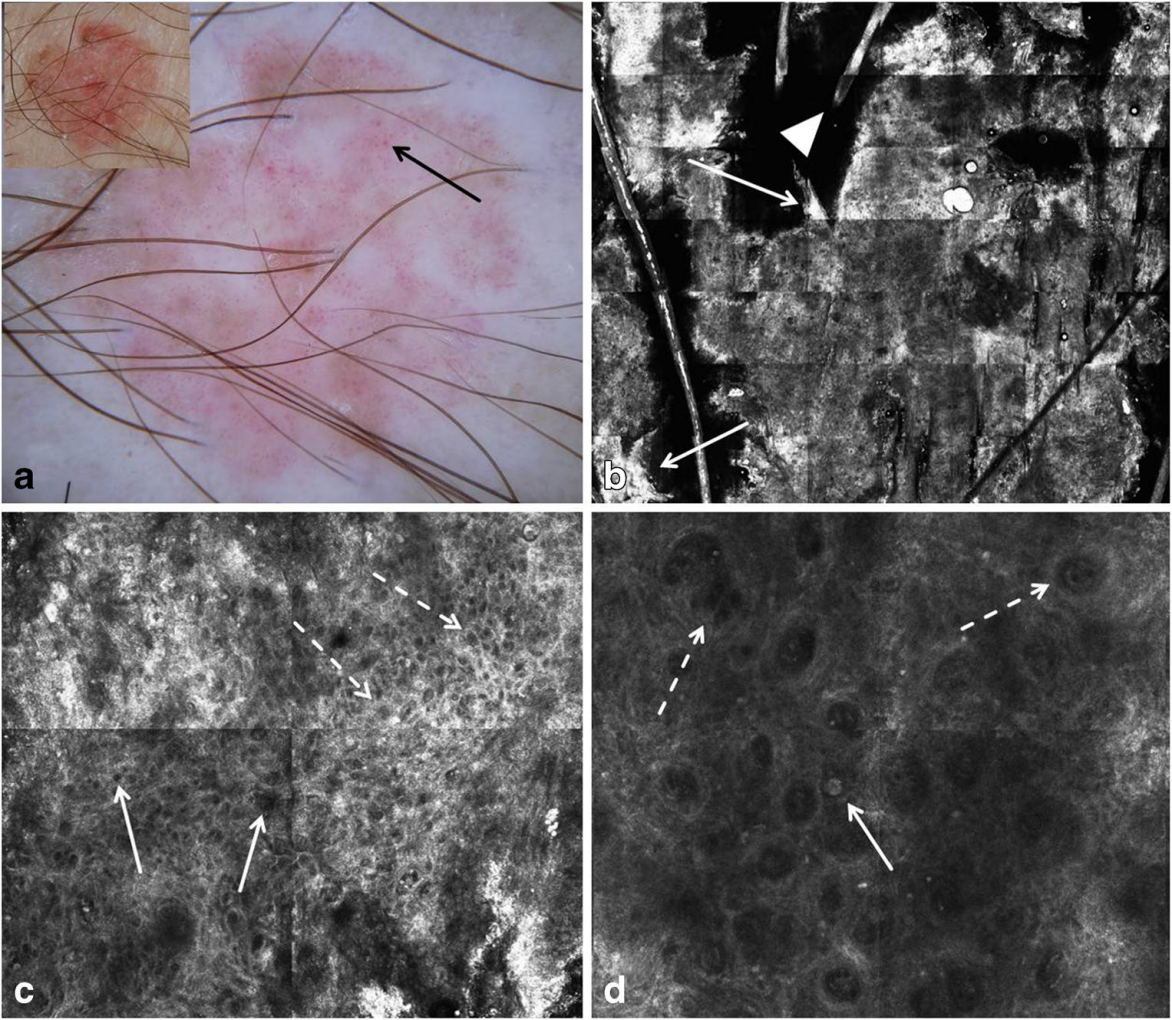
Clinical, dermoscopic, and RCM images of Bowen’s disease (SCC in situ). **a** Clinically, a red-brown scaly plaque measuring 15 mm in diameter is seen (inset). An ill-defined pink-brown structureless lesion with diffuse dotted vessels (arrow) is observed on dermoscopy. **b** On RCM at the level of the stratum corneum, irregularly shaped and heterogeneously bright sheets with loss of the normal skin folds are seen at low magnification (3.5 × 4 mm field-of-view). The brighter foci (arrows) correlate to the scale seen clinically. Hair shafts (arrowhead) are also seen. **c** An irregular honeycomb pattern is seen at the level of the spinous layer (1 × 1 mm field-of-view), with variability in the width and brightness of the lines (dashed arrows) and size and shape of the holes (arrows). In foci, large holes, representing abnormally large nuclei of keratinocytes are seen. **d** At the DEJ, increased density of dermal papillae having different sizes and shapes (dashed arrows) and harboring dilated blood vessels are visualized (1 × 1 mm field-of-view). In addition, a single large, round, bright-nucleated cell is seen (arrow) at the basal layer of the epidermis, probably correlating to a dyskeratotic keratinocyte

On RCM, SCC shows similar, albeit more pronounced findings compared to AK [6] (Fig. 2). Bright, round cells within the granular and spinous layers, whose nuclei are often obscured, are more frequently observed in SCC, correlating to dyskeratotic cells seen on histopathology. The honeycomb pattern is focally lost in the spinous and granular layers (“disarranged” pattern, Table 2). Dilated, round blood vessels (Table 2) running perpendicular to the horizontal confocal plane of imaging, in a looped or tortuous course, are observed within dermal papillae in most cases of SCC and less often in AK; these vessels may also be visualized as glomerular or dotted vessels on dermoscopy (Fig. 2a, d).

At present, RCM does not allow differentiation with high specificity between AK, SCC in situ and invasive SCC. It does, however, allow differentiation from other disease entities that occur on sun-damaged skin, such as BCC, solar lentigo, lichen planus-like keratosis, and melanoma, which is especially valuable in the facial skin [7]. Pigmented AK and SCC, however, represent potential diagnostic pitfalls because of overlapping dermoscopic and RCM features with lentigo maligna; the presence of gray dots in dermoscopy and of bright epidermal dendritic cells on RCM, corresponding to Langerhans cells, may result in an overdiagnosis of melanoma [8, 9].

The evaluation of genital skin diseases, including penile carcinoma in situ, is a new promising area of research in RCM [10].

A recent systematic review on the value of RCM for diagnosing actinic keratosis, actinic cheileitis, erythroplasia of Queyrat, Bowen disease, invasive SCC, and keratoacanthoma (KA) showed an overall range of sensitivity and specificity of RCM between 79 and 100% and 78–100%, respectively [11].

### Reflectance Confocal Microscopy of Basal Cell Carcinoma

**Fig. 3:**
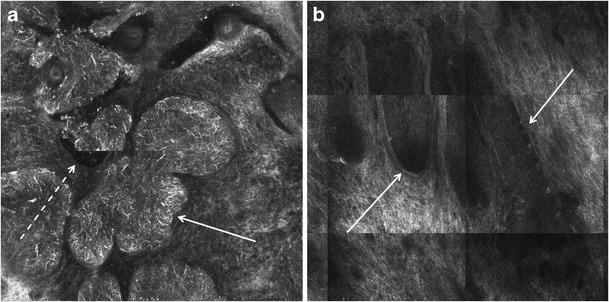
RCM image (1 × 1 mm field-of-view) of bright tumor islands and dark silhouettes seen in basal cell carcinoma (BCC). **a** On RCM, the bright tumor islands of BCC harbor numerous bright dendritic structures (arrow). These are dendrites of melanocytes in pigmented basaloid aggregates of BCC. Dark cleft-like spaces are also observed surrounding the tumor islands (dashed arrow). **b** Hyporeflective tumor islands appear as “dark silhouettes” (arrows) within the bright collagen of the tumor stroma; these correlate with non-pigmented basaloid aggregates of BCC

González et al. first described RCM features of BCC in 2002 [12]. The most striking feature of BCC with RCM is the presence of tumor islands (Table 2), sharply demarcated from the surrounding dermis by dark cleft-like spaces (Fig. 3). Bright tumor islands are typically seen in pigmented BCCs. The presence of tumor islands as hyporeflective areas (“dark silhouettes”) that are darker than the surrounding dermis or basal epidermis are mostly seen in non-pigmented BCCs (Fig. 3b). The tumor islands seen on RCM correlate on histopathology with aggregates of basaloid cells with peripheral palisading of nuclei and clefting. The bright and the darker tumor islands can also be seen in the same BCC reflecting the degree of pigmentation within the neoplastic aggregates. Bright dendritic structures and/or dendritic cells can be seen within refractile tumor islands and represent melanocytes in pigmented BCC (Fig. 3a); bright dendritic cells in the overlying spinous layer of the epidermis mostly represent Langerhans cells. Interestingly, when viewed by RCM at the *en face* plane, basal or spinous keratinocytes above tumor islands in superficial BCC appear to be focally elongated and distorted into alignment along an axis (streaming) (Table 2). The papillary dermis displays dense bright collagen bundles between the tumor islands, correlating to the fibrotic stroma associated with BCC. Dilated and tortuous linear blood vessels (Table 2) that run parallel to the horizontal plane of RCM imaging are also typically observed within the stroma. These vessels correlate with the arborizing or fine branching vessels seen on dermoscopy. In pigmented BCC, oval to triangular plump-bright cells with indistinct nucleus may be observed on RCM singly or in aggregates within the stroma; these correlate with melanophages on histopathology.

Subsequent studies validated the reliability of described RCM criteria of BCCs [13]; a recent meta-analysis of the diagnostic accuracy of RCM for BCCs showed a high summary estimate of sensitivity (97%) and specificity (93%) [14]. However, the number of infiltrative BCCs included in previous RCM studies was generally small, and the detection of this BCC subtype still remains challenging with RCM; the small cords and strands of neoplastic cells are difficult to detect within the bright dermal collagen. In addition, the limited depth of RCM imaging does not allow detection of more deeply infiltrating BCC.

## RCM of Melanocytic Neoplasms

RCM is a useful tool for the evaluation of melanocytic lesions since melanin is highly reflective and provides strong contrast in RCM images. RCM usually enables rapid recognition of pigmented skin lesion as melanocytic or as non-melanocytic; hypo- or amelanotic melanoma may show only slight reflectivity of melanocytes, but displays concordant cytologic and architectural abnormalities with pigmented melanoma. This is especially valuable in facial lesions, since the main differential diagnoses in pigmented and non-pigmented facial macules in the elderly is between lentigo maligna and non-melanocytic skin neoplasms such as solar lentigo/evolving seborrheic keratosis, lichen planus-like keratosis, basal cell carcinoma, and AK/SCC whose RCM criteria have been well defined [15].

In addition, the findings in RCM images allow differentiation of most nevi from melanoma. As the basis for RCM evaluation of melanocytic lesions, a publication has provided a consensus set of RCM terms [16], considering previously described RCM terms of melanocytic and non-melanocytic skin lesions; the glossary of terms was also accompanied by illustrative figures. Most of the terms used to evaluate melanocytic lesions showed high interobserver agreement among experts [17].

In addition, because RCM and dermoscopy both image lesions *en face*, RCM has been helpful in elucidating the direct tissue correlates of dermoscopic structures such as streaks or blue-white structures for which indirect correlation with the vertically sectioned histopathology is difficult. Dermoscopic and histologic correlates of RCM features have been described for nevi and melanoma [18, 19]. The value of RCM for the diagnosis of clinically and dermoscopically equivocal melanocytic neoplasms has been shown by a meta-analysis and is still the subject of ongoing investigation [20].

### RCM of Melanocytic Nevi

**Fig. 4:**
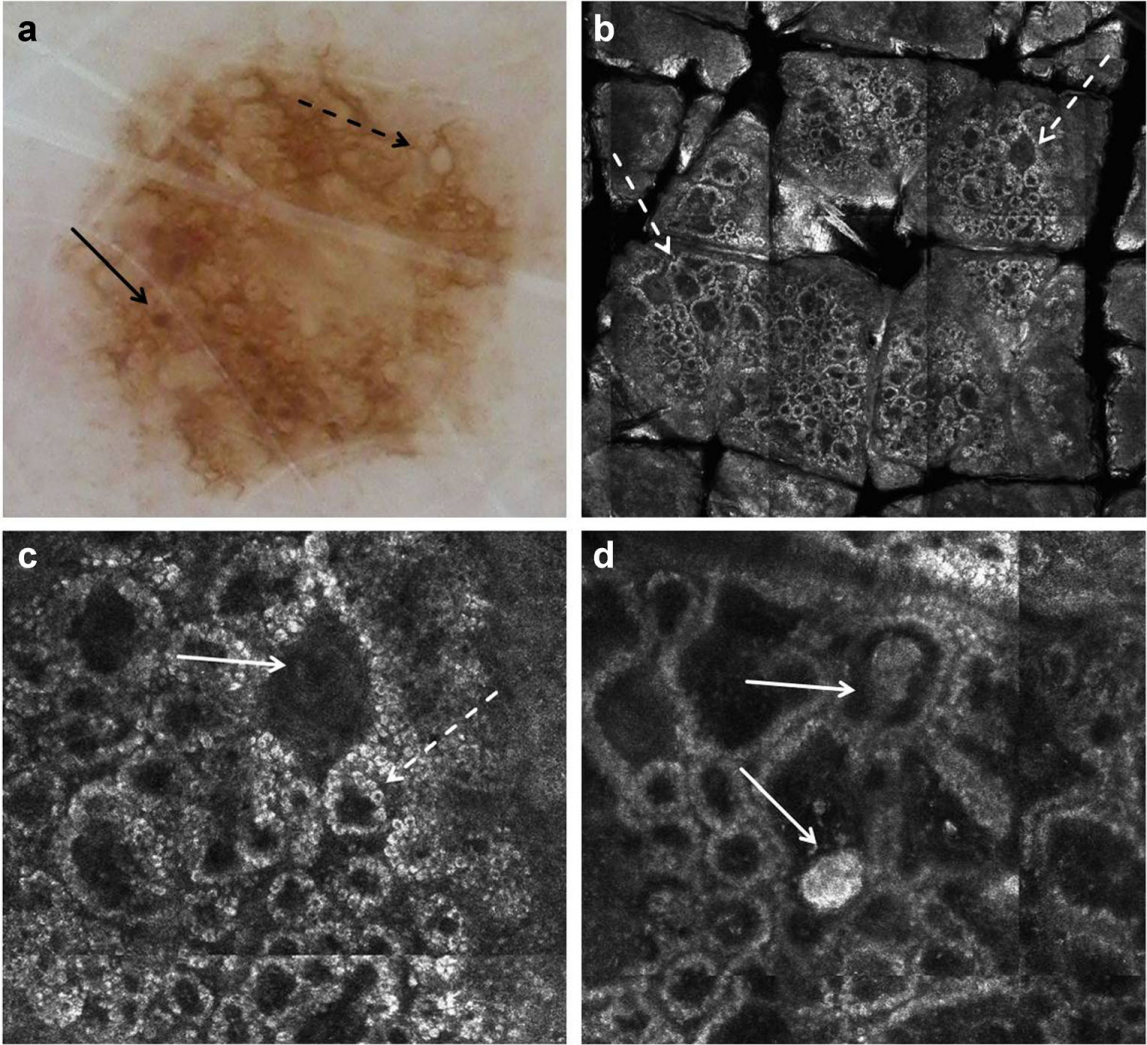
Dermoscopic and RCM images of a junctional nevus. **a** Dermoscopy shows a pigment network (dashed arrow) and focal small brown globules (arrow). **b** On RCM mosaic, a ringed pattern (arrows) composed of edged dermal papillae is observed at the DEJ (2 × 2 mm field-of-view). **c** At higher magnification (500 × 500 μm field-of-view), edged papillae are seen composed of dark round to oval dermal papillae (arrow) surrounded by a rim of monomorphous bright cells (basal keratinocytes and small melanocytes, dashed arrow). Round blood vessels traversing dermal papillae are detected during real-time imaging. **d** A deeper section of the DEJ displays small junctional dense nests (arrows) that protrude into the papillary dermis

**Fig. 5:**
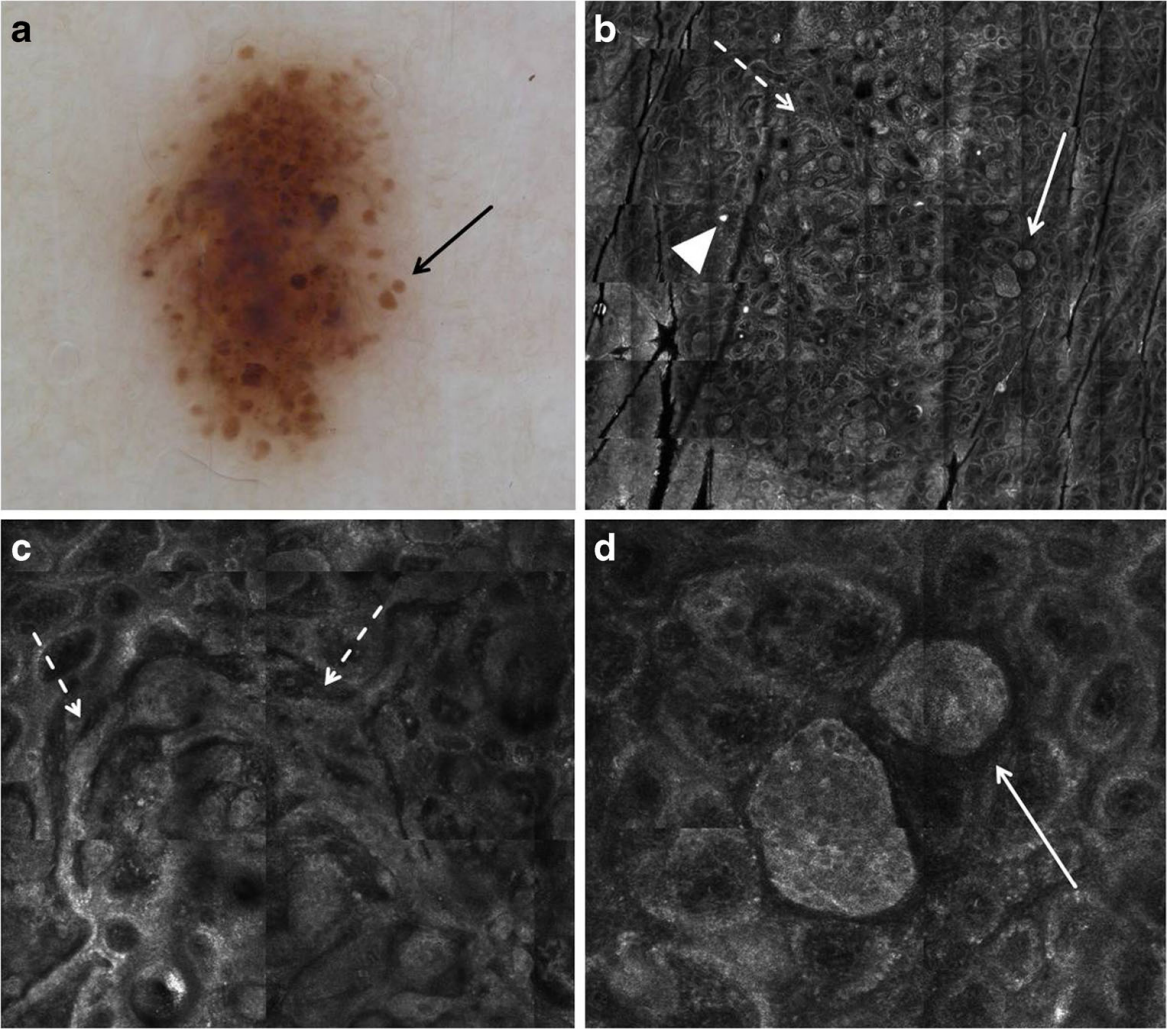
Dermoscopic and RCM images of a compound nevus. **a** Dermoscopy shows light to dark brown aggregated globules (arrow). **b** On RCM, a clod-and-meshed pattern is seen at the DEJ on low magnification (3 × 4 mm field-of-view); the clods being bright round structures (arrows) and the mesh being elongated, tubular structures (dashed arrow), both correlating to melanocytic nests. **c** At higher magnification (1 × 1 mm field-of-view), the tubular structures observed at the DEJ are junctional thickenings (dashed arrows) that widen and bridge the rete ridges. **d** Round to oval dense nests are seen within the dermal papillae on RCM (1 × 1 mm field-of-view); these are dermal nests

**Fig. 6:**
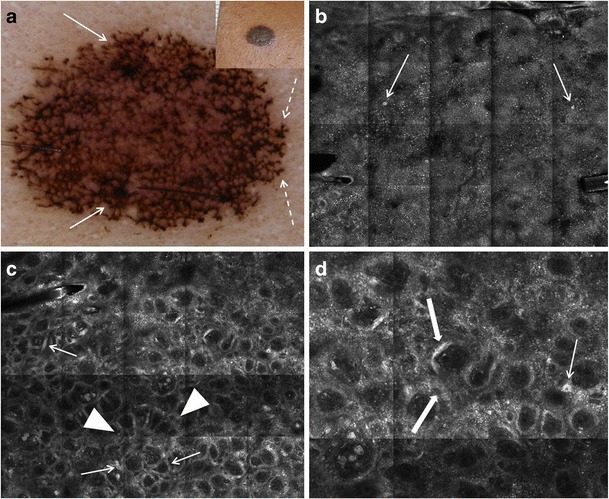
Images of a biopsy-diagnosed compound Clark (dysplastic) nevus, which shows features raising concern for melanoma on both dermoscopy and RCM. **a** Clinically (inset), a 4 × 5 mm dark brown, flat topped papule is seen. With dermoscopy, a dark brown atypical pigment network (arrows) and focal pseudopods at the lesion’s periphery (dashed arrows) are observed. **b** On RCM mosaic (2 × 2.5 mm field-of-view), few bright large cells (arrows) are seen at the level of the spinous layer, corresponding to melanocytes in Pagetoid pattern. **c** On RCM mosaic (2 × 2.5 mm field-of-view) at the DEJ level, a meshed pattern (arrowheads) is seen, composed of edged as well as non-edged papillae and bright tubular structures (“junctional thickening,” arrows); these tubular structures correlate with confluent melanocytic nests on histopathology. **d** Non-edged papillae are visualized on higher magnification RCM (approximately 1 × 1 mm field-of-view) at the DEJ level. Widened interpapillary spaces harboring bright tubular structures of variable reflectivity (“junctional thickening,” thick arrows) are observed. Single large, bright, nucleated cells are detected (arrow); these cells represent solitary atypical melanocytes. According to Pellacani’s RCM method for melanoma diagnosis, the lesion will get a score of 5 (non-edged papillae—2, atypical melanocytes at DEJ—2, cells in pagetoid pattern—1), denoting suspicion for melanoma

The RCM features of melanocytic nevi were first described by Langley et al. [21]. Nevi typically display round to oval, bright, monomorphic melanocytes, and a regular epidermal architecture. According to their dermoscopic and histopathologic appearance, different nevus types may show variable findings with RCM [22]. In junctional nevi, melanocytes are distributed singly and in nests mostly at the tips and sides of the rete ridges. With RCM, dark round dermal papillae surrounded by a rim of monomorphous refractive cells (“edged papillae,” Table 3), corresponding to melanocytes and pigmented basal keratinocytes, are observed at the level of the DEJ (Fig. 4b, c). Small junctional dense nests of melanocytes are observed on RCM within the interpapillary spaces or protruding into the dermal papillae with connection to the basal layer of the epidermis (Fig. 4d and Table 3). When viewing a mosaic image of nevi (akin to low magnification) at the DEJ level, the edged papillae, homogeneously distributed throughout the lesion, result in a regular “ringed pattern,” which correlates with typical pigment network seen on dermoscopy (Fig. 4a, b). In compound and dermal nevi, histopathology shows large nests of melanocytes within the dermis, with or without concomitant junctional nests. On RCM, compact aggregates of cells (also called dense nests), which are round to oval in shape and show uniform brightness, are observed (Fig. 5). Distinct cellular outlines of melanocytes are not always detectable within individual nests. When viewed on mosaic RCM image at the DEJ level, these large dermal nests may appear as a “clod pattern”; the larger nests of melanocytes seen with RCM correlate to pigmented globules seen with dermoscopy (Fig. 5a, b) Nevi may show on RCM at the DEJ level elongated, tubular melanocytic nests that expand the interpapillary spaces (“junctional thickening,” Table 3) (Fig. 5c); these correlate on histopathology with confluent junctional nests of melanocytes. When viewed on mosaic RCM image at the DEJ level, junctional thickening may appear as a “meshed pattern”; this correlates on dermoscopy with thickened network (Fig. 6).

**Table 3:** Key RCM features of melanocytic lesions

RCM feature	Definition	Favors melanoma or nevus?
Superficial epidermal layers (granular-spinous layers)		
Disarranged pattern	Focal or diffuse loss of the normal patterns of the granular-spinous layers (honeycomb or cobblestone). Often seen in conjunction with pagetoid spread of cells.	Melanoma > nevi
Pagetoid spread of cells	Presence of bright round or dendritic nucleated cells (melanocytes*) at suprabasal layers of the epidermis.	Melanoma > nevi
Dermo-epidermal junction		
Edged papillae	Dermal papillae demarcated by a rim of bright cells (pigmented basal keratinocytes and melanocytes).	Nevi > melanoma
Non-edged papillae	Dermal papillae without a demarcating bright rim at the DEJ; often associated with widening of the interpapillary spaces and with the presence of atypical melanocytes.	Melanoma > nevi
Dense nests	Compact, round to oval cell aggregates of variable reflectance in which outline of individual cells is often indiscernible. Junctional—the nests are connected with the basal layer of the epidermis and bulge into the dermal papillae. Dermal—the nests are located in the dermis without connection with the basal layer of the epidermis.	Nevi > melanoma
Junctional thickening	Enlargement of the interpapillary spaces (i.e., rete ridges) by bright cell aggregates. Outline of individual cells is often indiscernible.	Nevi > melanoma
Sheet-like distribution of cells	Round or dendritic nucleated cells (melanocytes) that are not aggregated in nests but closely distributed at the transition of the epidermis and dermis (DEJ) that shows loss of dermal papillae.	Melanoma > nevi
Superficial dermis		
Sparse nests	Aggregates of melanocytes with uneven brightness and cellular discohesion showing isolated nucleated cells at the periphery.	Melanoma > nevi
Cerebriform nests	Confluent aggregates of low reflecting cells in the dermis separated by a darker rim, resulting in a multilobate appearance.	Melanoma with dermal component

*Dendritic cells at the spinous layer of the epidermis occasionally prove to be Langerhans cells and not melanocytes. At present, this is a morphologic pitfall of RCM

Spitz nevi, some Clark nevi, and uncommonly congenital nevi, can present conflicting RCM criteria that may sway the observer to the diagnosis of melanoma. Concerning RCM findings that can be seen in these nevi include melanocytes at suprabasal layers of the epidermis (i.e., in pagetoid pattern); large solitary melanocytes at the basal layer; dermal papillae that are not well demarcated (known as “non-edged papillae,” Table 3); and melanocytic nests that vary in size, brightness and cohesiveness (including “sparse nests,” Table 3). Of note, these nevi are often also equivocal for clinical and dermoscopic diagnosis. In these nevi, RCM examination cannot rule out the possibility of melanoma [23•]. Thus, excisional biopsy and histopathological analysis are judicious in such concerning melanocytic lesions.

### RCM of Melanoma

**Fig. 7:**
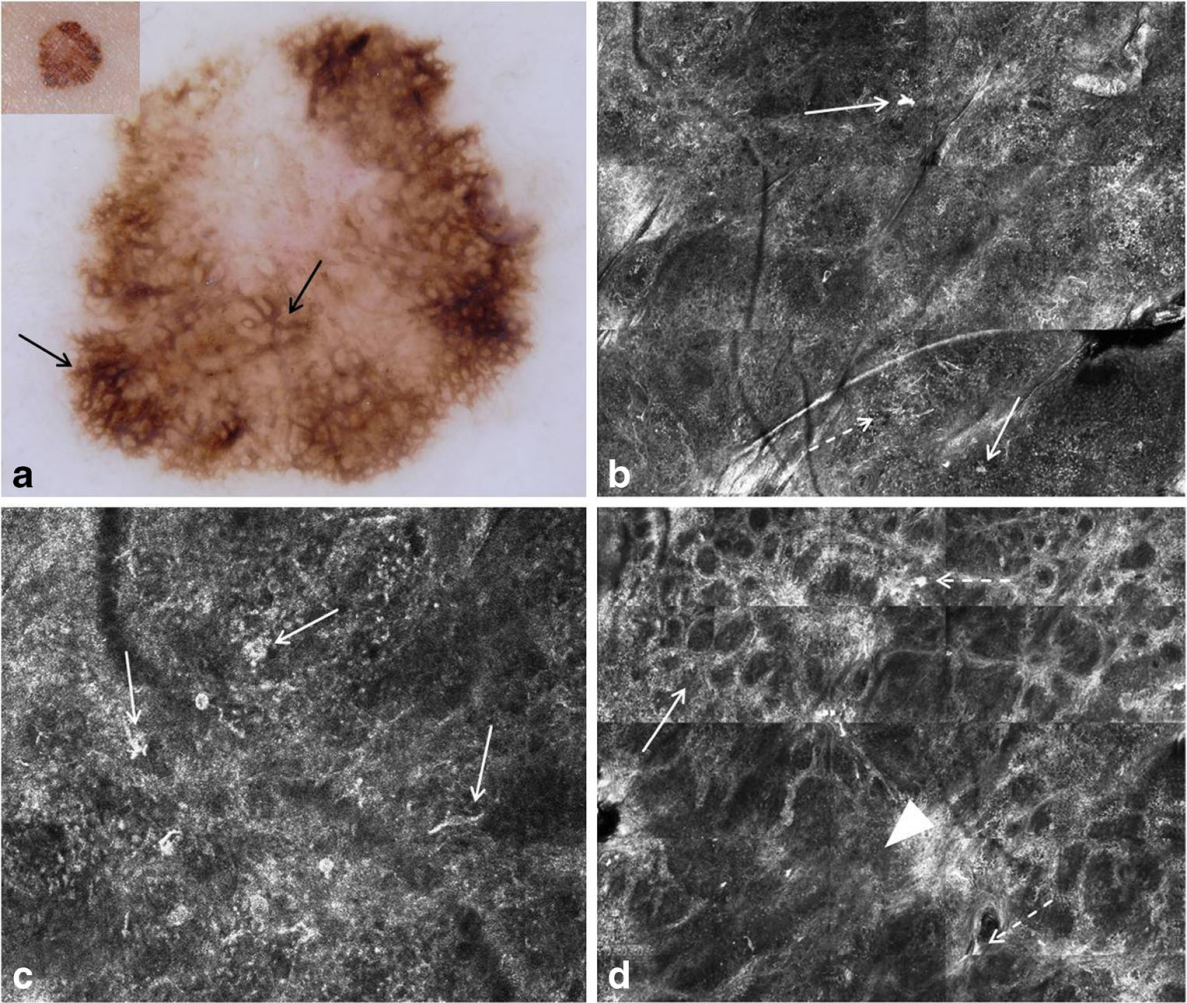
Clinical, dermoscopic, and RCM images of a melanoma in situ. **a** Clinically, a tan to dark brown patch with a diameter of 10 mm is seen. With dermoscopy, a dark brown atypical network (arrows) is observed. **b** With RCM mosaic (2.5 × 2.5 mm field-of-view), large, bright cells are seen within the upper epidermis (arrows). In addition, areas displaying bright dendritic processes are observed (dashed arrow). **c** Large, bright, nucleated cells of variable size and shape are seen at level of the spinous layer at higher magnification RCM (500 × 500 μm field-of-view), denoting melanocytes in Pagetoid pattern displaying pleomorphism. The normal honeycomb pattern is not seen (“disarranged pattern”). **d** On RCM mosaic (2.5 × 2.5 mm field-of-view) at the DEJ level, an irregular meshed pattern is seen (arrow) with some variability in the width and brightness of the lines of the meshwork. Focally, the meshed pattern is not seen (arrowhead); there is absence of dermal papillae in this focus, suggesting a flattened DEJ. Single bright cells (dashed arrows) stand out both within the meshwork and in the focus that lacks the meshed pattern, suggesting focal predominance of solitary melanocytes at the DEJ

**Fig. 8:**
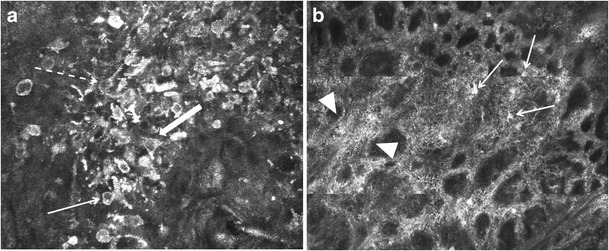
**a** RCM image (500 × 500 μm field-of-view) displaying pleomorphism of melanocytes in a melanoma. Round, triangular, dendritic, and spindle-shaped nucleated cells of variable size and reflectivity are seen at the suprabasal levels of the epidermis. There is a disarranged pattern, with loss of the normal honeycomb pattern. **b** RCM image (1 × 1 mm field-of-view) of a melanoma at the DEJ level displays junctional thickenings (arrowheads) with variable reflectivity and with widening of the interpapillary spaces. Multiple bright-nucleated and dendritic cells of variable size and shape (arrows), correlating to pleomorphism of melanocytes, are seen

**Fig. 9:**
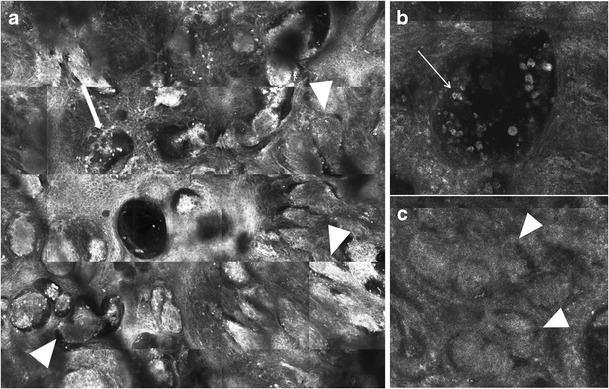
RCM images of a melanoma. **a** On RCM mosaic (2.5 × 2.5 mm field-of-view), multiple round, oval and tubular structures of variable reflectivity (arrowheads) are seen at the suprabasal epidermal levels; these correlate with nests of atypical melanocytes. The majority of the nests appear as “dense clusters” that are homogenous in brightness and in which individual melanocytes are mostly unapparent; however, “sparse nests,” which correspond to discohesive aggregates of atypical melanocytes, are also seen (arrow). **b** On high magnification RCM (500 × 500 μm field-of-view) of the upper epidermis, sparse nests appear as aggregates of nucleated cells of variable reflectivity, in which the individual cells are discernible. **c** High magnification RCM (500 × 500 μm field-of-view) at the level of the superficial dermis shows “cerebriform nests.” Cerebriform nests are confluent aggregates of low reflecting cells (melanocytes) in the dermis that are separated by a darker rim, resulting in a multilobate appearance

In melanoma, bright-nucleated cells (corresponding to atypical melanocytes) and dendritic processes may be seen at suprabasal layers of the epidermis (i.e., in pagetoid pattern) (Fig. 7). Melanocytes disposed in pagetoid pattern may be shaped as round, triangular, spindle cells, or dendritic nucleated cells [24]; melanocytes of different shapes, size, and brightness observed concomitantly suggest cellular pleomorphism (Fig. 8a). Clusters of melanocytes in pagetoid pattern may be observed, in addition to solitary pagetoid melanocytes. An abnormal epidermal pattern is usually seen; the honeycomb pattern is not visualized, either focally or throughout the lesion (“disarranged honeycomb pattern”) (Table 3 and Fig. 7c). At the DEJ level, an abnormal architecture is also detected with RCM. Non-edged dermal papillae (Table 3) variable in size and shape may be observed; non-edged papillae are often separated by widened interpapillary spaces that may contain large reflecting nucleated cells (atypical melanocytes) (Fig. 8b). While junctional thickenings (on high magnification RCM) and meshed pattern (on RCM mosaic images) can be seen at the DEJ in nevi and in some melanomas, their pattern is different. In nevi, the meshed pattern would appear more regular, while in melanoma, the junctional thickenings which contribute to the lines of the mesh, would vary in thickens and in brightness (Fig. 8b). In addition, large melanocytes as solitary units can be observed in melanoma at the basal layer of the epidermis (Fig. 8b). Foci with loss of the dermal papillae, replaced by sheets of atypical melanocytes seen on RCM (Table 3), indicate a crowded proliferation of solitary melanocytes and flattening of the DEJ on histopathology. In melanomas with a dermal component, cell clusters seen on RCM at the level of the superficial dermis may appear at least focally as sparse or cerebriform aggregates (Fig. 9b, c and Table 3). The variable size, shape and brightness, and uneven distribution of melanocytic nests seen in melanoma on RCM mosaics (Fig. 9a) correlate on dermoscopy with the presence of irregular globules and dots. The finding on RCM of cell clusters or solitary bright-nucleated cells in the dermis in a melanoma suggests the presence of an invasive melanoma. Dermal aggregates of plump-bright cells without visible nucleus (correlating with melanophages) as well as bright dermal clusters of melanocytes are seen in areas showing blue-white structures on dermoscopy.

Pellacani et al. defined six RCM criteria for the diagnosis of melanoma; two major criteria (non-edged papillae, atypical cells at the DEJ) and four minor criteria (roundish pagetoid cells, pagetoid cells widespread throughout the lesion, cerebriform clusters and isolated nucleated cells within dermal papillae) contribute to a scoring algorithm (each major criterion 2 points, each minor criterion 1 point). A score ≥ 3 signifies the diagnosis of MM with a sensitivity of 97.3% and a specificity of 72.3% [25]. A two-step algorithm for the diagnosis of melanoma by RCM has also been proposed [26]. In the first step, the lesion is identified by RCM as melanocytic or non-melanocytic. In the second step, the lesion is diagnosed with RCM based on Pellacani’s scoring system as nevus versus melanoma. By using this algorithm, unnecessary biopsies may be reduced by more than 50% without missing a melanoma.

The above mentioned RCM features are well established for superficial spreading melanoma. However, different melanoma subtypes may show distinct combinations of RCM features. For example, in nodular melanoma, cerebriform dermal nests are more commonly seen, while melanocytes in pagetoid pattern or a disarranged honeycomb pattern of the epidermis are uncommon [27].

**Fig. 10:**
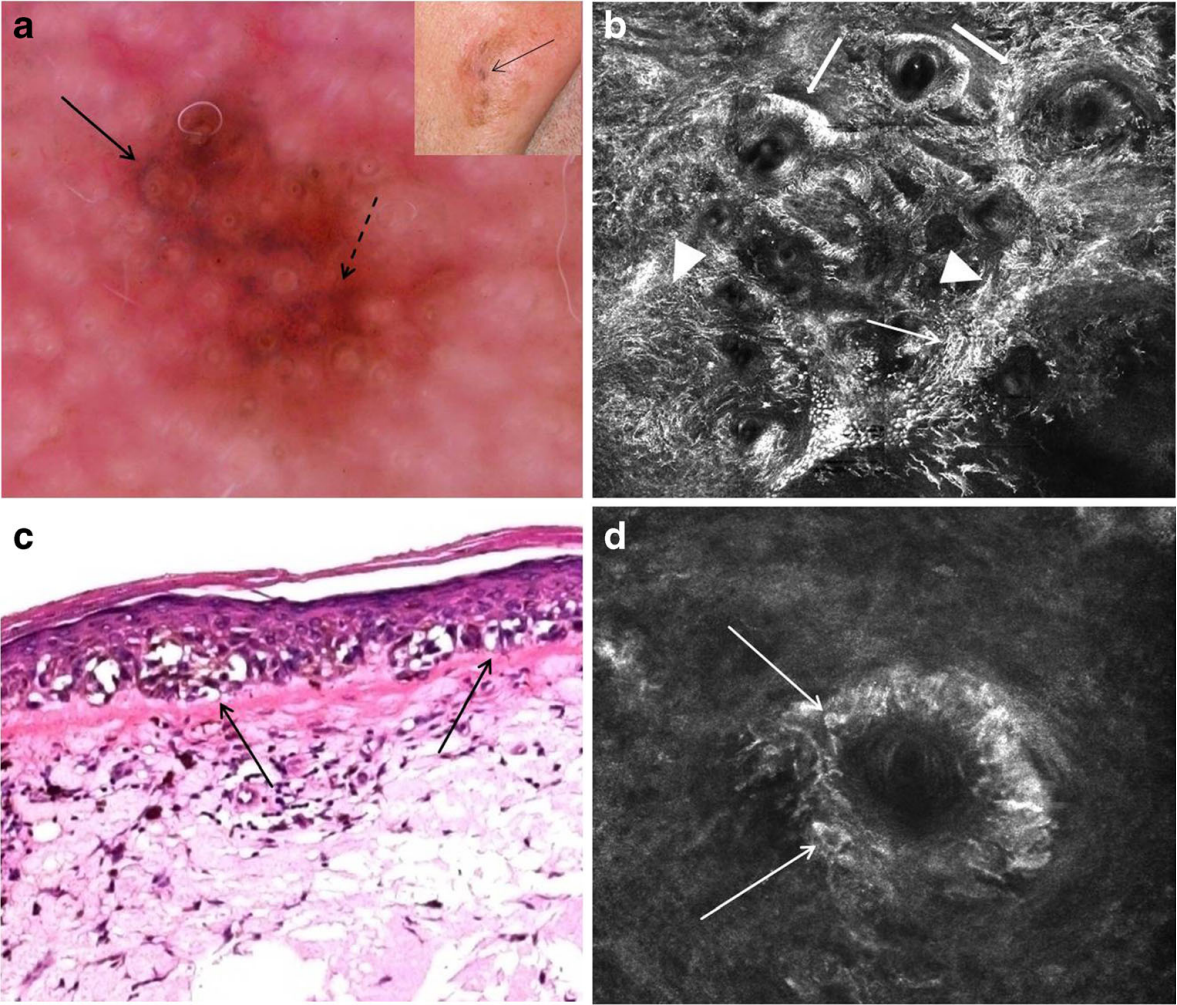
Clinical, dermoscopic, histopathological, and RCM images of a facial melanoma in situ (lentigo maligna). **a** Clinically (inset), a 2 × 3 cm tan macule with focal dark brown areas is seen. With dermoscopy, asymmetrically pigmented follicular openings (arrow) and early rhomboidal structures (dashed arrow) are visualized. **b** On RCM (approximately 2 × 2 mm field-of-view), bright dendritic cells and dendritic processes (arrowheads) form sheets and tubular structures at the basal layer. Small melanocytic nests are focally detected (thin arrow). Some of the sheets and tubular structures of dendritic cells and dendritic processes are located around adnexal structures (thick arrows). **c** On histopathology, atypical melanocytes are seen as crowded single cells and small nests (arrows) along a flattened DEJ (H&E, magnification × 100). **d** At high magnification RCM (500 × 500 μm field-of-view), bright, pleomorphic nucleated, and dendritic cells infiltrate the adnexal epithelium (arrows); imaging deeper into the tissue can show the extension of atypical melanocytes down adnexal structures

In melanoma on sun-damaged skin (lentigo maligna), a descent of atypical melanocytes along adnexal structures, aggregates of atypical melanocytes surrounding adnexal openings, sheets of atypical (predominantly dendritic) melanocytes and dendrites at basal/suprabasal layers, and cord-like rete ridges at the DEJ are typically observed [28]. A so-called ringed pattern is usually not observed due to the flattening of the DEJ in sun-damaged skin of the elderly (Fig. 10 and Table 3). The value of RCM as adjunct to dermoscopy in the diagnosis of skin neoplasms in this cosmetically sensitive area has repeatedly been shown; RCM correlates of dermoscopic patterns of facial lesions help discriminating lentigo maligna from pigmented non-melanocytic macules [29•].

Studies have demonstrated the potential benefit of RCM in the differentiation of benign pigmented genital skin lesions from melanoma, including nevi and melanotic macules [30]. The presence of bright epidermal dendritic cells, corresponding to Langerhans cells, in a considerable number of melanotic macules represented a possible diagnostic pitfall in the differentiation from melanoma.

Finally, RCM is particularly valuable in the evaluation of lightly pigmented or amelanotic melanomas that often lack diagnostic features on clinical and dermoscopic examination [31].

Limitations of RCM in the assessment of melanocytic skin lesions include the lack of nuclear staining, the limited depth of imaging, and the difficulty in distinguishing dendritic melanocytes in pagetoid pattern from Langerhans cells that may occasionally simulate pagetoid spread.

## RCM in Margin Mapping and Monitoring of Treatment of Skin Cancer

There have been several attempts to use RCM in the follow-up of skin cancer treatments. Among others, the ability to confirm tumor clearance after cryotherapy, topically applied imiquimod, and ingenol mebutate gel or in patients receiving Vismodegib has been assessed for NMSC [32, 33]. The value of RCM in the monitoring of melanoma after therapy with topically applied imiquimod, as well as for presurgical margin mapping of clinically difficult to delineate skin cancers, has been evaluated. However, these studies consisted mostly of small case series [34].

Another focus of research lies in the use of RCM in microscopic-guided surgery, such as Mohs surgery. In previous studies, contrast agents, which led to compaction of chromatin, such as acetic acid, were used to improve imaging contrast. However, micronodular and infiltrative BCCs were not consistently identified via RCM within the background of bright dermal collagen, and the detection of SCCs was hindered by the bright appearance of the epidermis. The recent introduction of fluorescence confocal microscopy, using fluorophores such as acridine orange as contrast agents, may overcome these drawbacks; an overall sensitivity and specificity of 96.6 and 89.2%, respectively, was reported for the ex vivo detection of BCCs, across all subtypes, in tissue specimens from Mohs surgery [35].

## Challenges and Future Development

Barriers to the adoption of RCM technology are the black and white contrast imaging and limited penetration depth. The rule of thumb of optical imaging is that the deeper it penetrates, the lower the resolution. To this end, use of multi-wavelength RCM may allow incorporation of wavelengths that penetrate deeper as well as wavelengths that penetrate more superficially but with higher resolution; assignment of pseudo-colors to images is also a possibility with multi-wavelength imaging, because the same structures may reflect differently at various wavelengths. As key RCM features of skin neoplasms are increasingly recognized, RCM is expected to significantly improve our diagnostic ability. However, at present, RCM does require expertise and experience in image interpretation, while image acquisition is fairly straightforward. The inherent potential of RCM for tele-dermatologic application and automated image analyzing can address the end-user interpretation challenges. Finally, in the future, a multimodal imaging concept combining RCM with other non-invasive imaging tools (like optical coherence tomography) may be applied to improve non-invasive imaging. The ongoing research in this field may also allow future expansion of the use of RCM in a broader variety of skin diseases, such as inflammatory skin diseases. The possibility to follow cytological and architectural changes in the same skin lesions over a longitudinal period offers a new dimension for the examination of the biological behavior of “borderline” melanocytic nevi and will help elucidating controversies in nevogenesis [36•].
